# Research of time processing disorder within the investigation of specific traits of schizophrenia

**DOI:** 10.1192/j.eurpsy.2022.640

**Published:** 2022-09-01

**Authors:** I. Szendi Md Habil, S. Szalóki, A. Bagi, E. Rudics, E. Hallgató

**Affiliations:** 1 Kiskunhalas Semmelweis Hospital, University Teaching Hospital, Psychiatry Unit, Kiskunhalas, Hungary; 2 University of Szeged, Department Of Psychiatry, Szeged, Hungary; 3 University of Szeged, Institute Of Psychology, Szeged, Hungary

**Keywords:** subjectivity, schizophrenia-bipolar spectrum, interval discrimination, interval production

## Abstract

**Introduction:**

Schizophrenia is essentially related to one’s self-perception and the relationship to the world. One possible explanation for symptoms of schizophrenia in activities is the disruption of timing, which can develop into a disorder of activity perception and attribution.

**Objectives:**

Our study aimed to investigate the specificity of time perception disorder within the schizophrenia-bipolar spectrum, within the time interval around one second.

**Methods:**

In the study, N = 15 schizophrenic (M = 37.28 years, SD = 9.49 years), N = 9 bipolar (M = 49.44 years, SD = 8.48 years), N = 10 schizoaffective (M = 41.32 years, SD = 10.75 years) patients with compensated clinical condition and N = 28 healthy control subjects (M = 36.5 years, SD = 9.9 years) participated. Time processing was examined with a perceptual (discrimination) and a productive (synchronization) task.

**Results:**

Concerning the interval discrimination, patients with schizophrenia and schizoaffective disorder lag behind controls in the majority of indicators (0.373–0.772). In terms of production and reproduction, the deviation of schizoaffective patients indicates a moderate difference, but subjects with schizophrenia show a large effect size, and subjects with bipolar disorder demonstrate a small effect size.

**Conclusions:**

Our results suggest that the schizophrenic group exhibits a comprehensive time-processing disorder and in this respect can be distinguished from the bipolar affective and the control group. People with schizoaffective disorder show an intermediate performance in reproduction between the schizophrenic and bipolar groups, while in the case of discrimination deficit, they approach schizophrenics.

**Disclosure:**

No significant relationships.

